# Examining the gender imbalance in the National Community Health Assistant Programme in Liberia: a qualitative analysis of policy and Programme implementation

**DOI:** 10.1093/heapol/czac075

**Published:** 2022-09-07

**Authors:** Sali Hafez, Bob Mwiinga Munyati, Katie Zeno, Catherine K Z Gbozee, Mbalu Jusu, Mantue S Reeves, C Sanford Wesseh, S Olasford Wiah, Mohammed AlKhaldi, Kristin Johnson, Marion Subah

**Affiliations:** Department of Public Health and Policy, London School of Hygiene and Tropical Medicine, 9 Tavistock PI, London WC1H 9SH, United Kingdom; Department of Public Health and Policy, London School of Hygiene and Tropical Medicine, 9 Tavistock PI, London WC1H 9SH, United Kingdom; American University School of International Service, 4400 Massachusetts Avenue NW, Washington, DC 20016, United States; Cuttington Graduate School of Professional Study and Health System Strengthening Unit, Last Mile Health Liberia, 77C6+984, Monrovia, Liberia; Department of Public Health and Policy, London School of Hygiene and Tropical Medicine, 9 Tavistock PI, London WC1H 9SH, United Kingdom; Mekelle University and the Monitoring, Evaluation and Research Unit, Last Mile Health Liberia, 77C6+984, Monrovia, Liberia; The Department of Health and Vital Statistics, the Ministry of Health, SKD Boulevard, Congo Town 1000, Monrovia, Liberia; Director, Community Health Services Division, the Ministry of Health, SKD Boulevard, Congo Town 1000, Monrovia, Liberia; Faculty of Medicine, School of Physical and Occupational Therapy, Person-Centred Health Informatics Research-PCHI lab, McGill University, 3605 Rue de la Montagne, Montréal, QC H3G 2M1, Canada; Department of Public Health, Boston University, Boston, MA 02215, United States; Department of Nursing, Catholic University of America, United States and Country Director, Last Mile Health Liberia, 77C6+984, Monrovia, Liberia

**Keywords:** Community health workers, gender, gender equity, gender assessment, health workforce, health policy, Liberia, gender responsiveness

## Abstract

The Revised National Community Health Services Policy (2016–2021) (RNCHSP) and its programme implementation, the Liberian National Community Health Assistant Programme (NCHAP), exhibit a critical gender imbalance among the Community Health Assistants (CHAs) as only 17% are women. This study was designed to assess the gender responsiveness of the RNCHSP and its programme implementation in five counties across Liberia to identify opportunities to improve gender equity in the programme. Using qualitative methods, 16 semi-structured interviews were conducted with policymakers and 32 with CHAs, other members of the community health workforce and community members. The study found that despite the Government of Liberia’s intention to prioritize women in the recruitment and selection of CHAs, the planning and implementation of the RNCHSP were not gender responsive. While the role of community structures, such as Community Health Committees, in the nomination and selection of CHAs is central to community ownership of the programme, unfavourable gender norms influenced women’s nomination to become CHAs. Cultural, social and religious perceptions and practices of gender created inequitable expectations that negatively influenced the recruitment of women CHAs. In particular, the education requirement for CHAs posed a significant barrier to women’s nomination and selection as CHAs, due to disparities in access to education for girls in Liberia. The inequitable gender balance of CHAs has impacted the accessibility, acceptability and affordability of community healthcare services, particularly among women. Strengthening the gender responsiveness within the RNCHSP and its programme implementation is key to fostering gender equity among the health workforce and strengthening a key pillar of the health system. Employing gender responsive policies and programme will likely increase the effectiveness of community healthcare services.

**Key messages**

- Despite the Government of Liberia’s intention to prioritize women in the selection of Community Health Assistants (CHAs) in the National Community Health Services Policy (NCHSP), the planning and implementation of the National Community Health Assistant Programme (NCHAP) resulted in a CHA workforce, i.e. majority men.
- The role of community structures in the nomination and selection of CHAs is central to community ownership of the programme. However, cultural and traditional perception of gender, gender norms and dynamics influenced the NCHAP implementation, which led to the inequitable gender balance of CHAs.
- Women’s limited access to education was a major barrier to their nomination and selection as CHAs. Cultural, social and religious practices, perceptions and norms of gender represented significant barriers to a gender-responsive NCHAP. Therefore, the Revised National Community Health Services Policy and NCHAP should further utilize a multisectoral approach to work with diverse stakeholders in Liberia to address societal barriers that influence the gender responsiveness of community health programming.
- Gender inequity in the Revised National Community Health Services Policy affected Liberia’s access, acceptability and affordability of healthcare services, particularly among women.

## Introduction

### Background

The Government of Liberia’s National Community Health Assistant Programme (NCHAP) has made enormous strides towards making Universal Health Coverage (UHC) a reality in Liberia. The programme, institutionalized through the Revised National Community Health Services Policy (RNCHSP) of 2016, extends the reach of Liberia’s primary healthcare system to remote communities through a cadre of community health workers (CHWs) known as Community Health Assistants (CHAs) (MOH, 2015). The CHAs are skilled, supervised, salaried and supplied and use a community-based information system (MOH, 2015; Healey *et al.*, 2021). However, gender inequity among CHAs remains a key obstacle in maximizing the NCHAP’s impact on the communities’ access to health services and programme sustainability, with women comprising just 17% of the CHAs employed by the programme since 2016 (Lisgis, 2021). Anecdotal evidence suggests that CHAs who are women are likely to continue working in the NHCAP programme compared with men, suggesting their impact on the programme sustainability.

A key goal of the RNCHSP was to improve health outcomes for women and children. The disease burden in Liberia disproportionately affects women and children, with maternal and under five mortality and access to family planning as leading public health challenges. According to the Demographic and Health Survey 2019–2020, Liberia has one of the highest maternal mortality rates globally at 742 deaths per 100 000 live births (Lisgis, 2021). The causes of maternal deaths are primarily attributable to preventable and treatable complications (MOH, 2016). In addition, the total fertility rate of 4.2 children per woman remains higher than the global average of 2.4 children per woman (MOH, 2016; Lisgis, 2021). From 2013 to 2019, the percentage of women whose family planning needs went unmet increased from 31.1 to 33.4 (Lisgis, 2013; 2021). While infant mortality rates decreased substantially from 1986 to 2007, they increased from 54 deaths per 1000 live births in 2013 to 63 deaths per 1000 live births in 2019 (Lisgis, 2021).

Many structural, economic and social barriers restrict access to essential healthcare in Liberia. Structural challenges of the health system include inequitable service coverage, inadequate human resources for the health workforce, insufficient infrastructure, poor supply chain management, limited availability of essential medical commodities and weak governance structures (Simen-Kapeu *et al.*, 2021). According to the Global Financing Facility (GFF) Investment Case, cost and distance to care are also major barriers to accessing essential health services (Moh, 2016). Delays in seeking care, gender dynamics, religious or cultural beliefs and fear of or lack of trust in the health system are additional barriers to accessing healthcare (Chen, 2019, Zeno, 2021).

In 2014, the Ebola epidemic hit Liberia and exacerbated the vulnerabilities of the health system. In addition to the devastating loss of life caused by the epidemic, there were significant disruptions in the delivery and utilization of health care services (UNDP, 2014). Community Health Volunteers (CHVs) and other frontline health workers played an essential role in detecting and tracing cases in remote communities (MOH, 2016). However, the Ebola epidemic also exposed the inefficiencies and gaps within the existing CHV programme established through the National Policy and Strategy on Community Health Services in 2008. Although community health models have operated in Liberia since establishing the primary healthcare programme in the 1978 Alma Ata Declaration, the Ebola epidemic reinforced the importance of investing in a standardized, formalized community health model. At the end of the epidemic, the Government of Liberia and its partners showed unprecedented political will to build a resilient health system with the intention of removing barriers to care for all Liberians (MOH, 2015).

### Community health workforce in Liberia

Historically, the community health structure in Liberia was highly fragmented, with several cadres of CHVs, including general Community Health Volunteers (gCHV), trained traditional midwives (TTM) and health promoters. The majority of the gCHVs were men, while all TTMs were women. However, the 2016 RNCHSP established an integrated supervisory cadre of Community Health Services Supervisors (CHSS) who are primarily professional nurses, midwives and physician assistants (MOH, 2015). The reformed policy shifted from CHVs to an incentive-based model, with formal remuneration of all CHAs. Further, CHAs are chosen by their community through Community Health Committees (CHCs) and provide a standard package of preventative, curative and promotive services (MOH, 2015). These services include integrated community case management for malaria, acute respiratory infection, diarrhoea, health promotion, community events-based surveillance and reproductive, maternal, neonatal and child health (MOH, 2015).

**Figure 1.**
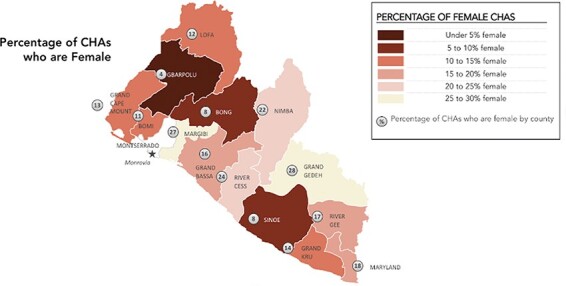
Gender distribution of CHAs in Liberia, 2020

The goal of the revised model was to recruit primarily women to become CHAs to improve the social, political and economic status of women in Liberia (GOL, 2012). Despite these aspirations and a written preference within the policy for women to become CHAs, currently, only 17% of CHAs and 49% of CHSSs are women (as shown in Figure 1) (Lisgis, 2021; NCHAP, 2021). This is inconsistent with evidence showing that globally, women make up the majority of CHWs (Masis *et al.*, 2021).

Liberia’s unique CHA gender imbalance may be because the policy and programme failed to include explicit guidance on operationalizing these recommendations, resulting in an inequitable recruitment and selection process of CHAs. For example, the CHA selection criteria favoured men who had high levels of education.

Further affecting the gender imbalance, the RNCHSP does not recognize TTMs as a formal cadre. While TTMs may receive compensation from the communities they serve or in one-off project-specific initiatives, remuneration is not mandated through the policy and they are not entitled to monthly compensation by the MOH or implementing partners (MOH, 2015). With community health services targeted mainly at women and children, the impacts of a male skewed CHA cadre have created unintended challenges in health services’ access, utilization and acceptability.

At the time of the study, the Government of Liberia was revising the RNCHSP that guides the NCHAP after its first five years of implementation. In tandem, the Ministry of Health (MOH) utilized this window of opportunity to conduct this comprehensive gender assessment of the RNCHSP and its programme implementation (NCHAP). The purpose of this study is to examine the gender responsiveness of the policy and programme, identify and document gender inequities that exist within the programme, such as the inequitable ratios of CHAs, assess whether and how the programme has impacted genders differently and provide recommendations for actionable policy revisions to strengthen gender responsiveness in the RNCHSP and NCHAP.

## Methods

### Study design

The study employed qualitative methods to collect primary data collection through 48 semi-structured in-depth interviews conducted between 17th August and 16 September 2021 in five counties of Liberia. As illustrated in Table 1. To inform developing the interview guides, a literature review was completed on gender, community health programme and the health workforce in Liberia and similar contexts. This study used the socioecological model to explore factors affecting the gender imbalance in the RNCHSP and NCHAP (Steege *et al.*, 2020) (Dahlberg and Krug, 2006).

**Table 1.** Qualitative interviews by participant type and location

Phase I—key informant interviews (total = 16)					
MOH	International organizations	National NGOs	United Nations	Donors	Ministry of Internal Affairs
4 x Men	1 x Man	1 x Man	1 x Man	2 x Women	1 x Woman
2 x Women	2 x Women	1 x Woman	1 x Woman		
**Phase II—in-depth interviews (total = 32)**					
Grand Cape Mount	Margibi	Nimba		Rivercess	Sinoe
CHFP 1 x Man	CHFP 1 x Man	CHSS 1 x Woman		HFDC 1 x Man	TTM 1 x Woman
HFDC 1 x Man	CHSS 1 x Woman	TTM 1 x Woman		CHA 1 x Man	CHC 1 x Woman
CHSS 1 x Woman	CHA 1 x Woman			CHSS 1 x Man	CHFP 1 x Man
TTM 1 x Woman	CHC 1 x Man	HFDC 1 x Man		OIC 1 x Man	CHA 1 x Woman
CHC 1 x Man	TTM 1 x Woman	CHFP 1 x Man		CHFP 1 x Man	CHSS 1 x Woman
CHA 1 x Man	HFDC 1 x Man	CHA 1 x Man		CHC 1 x Man	OIC 1 x Man
		Patient 1x Woman		HFDC 1 x Man	
				Patient 1 x Woman	

This paper comprehensively examines the gender responsiveness of the RNCHSP and its implementation in two phases. The study defines gender responsiveness as ‘the planning process in which programme and policy actions are developed to deal with and counteract problems which arise if the socially constructed differences between women and men are not adequately addressed’ (UNDP, 2015). Phase I of the study investigated the gender responsiveness of the RNCHSP to identify the strengths, weaknesses, barriers and enablers that influenced the inequitable gender ratio of the community health workforce and how the policy influenced the gender responsiveness of the NCHAP’s design and implementation. Phase II focused on investigating the root causes of gender inequity in the NCHAP, whether and how the programme impacted genders differently, including access and quality of care and how gender dynamics at the household and community levels influenced the inequitable gender ratio of CHAs in the NCHAP. Finally, the finding informed actionable recommendations for the policy and programme.

The study was guided by an expert advisory panel, including the MOH and organizations working on gender and community health policy and programme in Liberia. The panel validated the study methods and tools to accurately reflect the political, cultural and gender in the Liberian context.

### Study participants and settings

Guided by the expert panel, the study participants were purposively sampled and selected according to their past, current or future engagement with gender and the RNCHSP, NCHAP, community health services or affiliation to a community in one of the study locations. Phase I of the study was conducted remotely with Monrovia-based respondents, referred to as ‘key informants’. They represented diverse policy actors engaged in community health, gender and the health workforce, including representatives from the Liberia MOH, Liberia Ministry of Internal Affairs, United Nations (UN) agencies, international nongovernmental organizations (INGOs) and local nongovernmental organizations (NGOs).

Phase II gathered the perspectives of NCHAP actors and the community members. The study team purposively sampled interviewees from the five counties of Sinoe, Rivercess, Nimba, Margibi and Grand Cape Mount, referred to as ‘study participants’. The county selection is based on three criteria: (1) counties with active NCHAP programme, (2) availability of county-based NCHAP programme staff and implementing partners and (3) representation across social, traditional, religious and cultural practices and norms. Phase II interviews were conducted with members of CHCs and other community structures, such as Health Facility Development Committee (HFDC) members, CHAs, CHSSs, TTMs, patients, civil society and community members.

### Data collection and analysis

In Phase I, the study team, as described in Appendix 1, conducted 16 one hour remote key informant interviews (KIIs). 56% of the Phase I study participants were women. In Phase II, a local researcher conducted face-to-face interviews of approximately 30–45 minutes each with 32 participants (40% women), representing the community’s perspective of the NCHAP implementation. The study team trained the local researcher on Phase II study tools to ensure data integrity and internal validity. The study tools were piloted and minor language changes were made. Additionally, a data management plan that incorporated data security, protection, anonymization and confidentiality of study participants was developed for both Phases I and II.

All the interviews were audio-recorded, transcribed and analysed. The study employed thematic analysis to identify overarching themes for each phase (Braun and Clarke, 2014). Data were coded using a mixed inductive and deductive approach to examine the assumptions and identify emerging themes (Clarke and Braun, 2018). A Liberian researcher reviewed the transcripts that were generated through auto-transcription software (Otter) to ensure their accuracy and context-specific expressions were noted and explained by the researcher. The study team developed a codebook and used digital text analysis software (MaxQDA) to code and construct the analysis of the KIIs. Independent members of the study team cross-reviewed each phase’s coding to ensure internal validity. External validity was examined through validation workshops with the advisory expert panel and a wider group of stakeholders of MOH, implementing partner organizations, CHAs from various counties, UN agencies and key donors. The validation confirmed the accurate interpretation of the data within the Liberian context and identified areas for additional research.

The socioecological model was used to structure the analysis, interpret the findings, develop recommendations and explain the validated findings. The study findings informed the development and selection of evidence-based recommendations for the new RNCHSP policy.

### Ethics

The study was examined and approved (protocol number 21-08-279) by the University of Liberia Pacific Institute for Research and Evaluation Institutional Review Board on 4 August 2021. The study’s purpose, potential benefits and risks were shared with participants and written or recorded verbal informed consent was obtained.

## Results

### NCHAP policy design process

The interviews showed that while policymakers intended to develop a gender-responsive community health services policy and programme by prioritizing the selection of CHAs who are women, these intentions fell short. Most study participants from the first phase of data collection revealed that the RNCHSP was designed as a standalone health policy to address health needs with limited considerations for the multisectoral factors that influence health. The policy design did not take into consideration the community dynamics, gender and power relations or structural, cultural and educational barriers that women in Liberia face, as illustrated discussed in ‘Methods’ section. Moreover, it did not consider the gendered consequences—such as the workforce gender imbalance—the policy could have on the community health workforce, as illustrated in ‘Results’ and ‘Discussion’ sections. By not designing the policy with a gender lens, the policy, when implemented, resulted in an inequitable gender ratio among CHAs.

So, I think there was an intent to be gender responsive, but I think it fell short when it came to implementation. KII-INGO (Woman)

The interviews confirmed the centrality and leadership of the MOH in the policy design. Donors, national organizations and key line ministries (e.g. the Ministry of Gender and Social Protection, Ministry of Interior Affairs, Ministry of Education, and Ministry of Youth and Sports) were engaged at the last stage of review, instead of employing a codesign approach. Thus, the opportunity for key gender actors to guide a gender-responsive policy design was limited. The result was a policy that did not provide a clear road map on how to implement it in a gender-responsive manner, including defining specific roles and areas of complementarity for the non-health actors.

(Gender). unfortunately, has not been directly part of their last review, but I have some information to increase percentage of women, that is still being pushed for, because we know that women are the one that are caregivers and every programme needs them to succeed, we need to have more women because they are the ones that remain in a community most of the time

Study participants believed that utilizing of a multisectoral approach in the policy design could have improved health outcomes, optimized financial and human resources of the different sectors and addressed the social determinants of health that are rarely addressed in health only policies.

The RNCHSP 2016–2021 represented a transforming milestone for community health in Liberia, however, the interviewees highlighted that the policy design process was challenged by the limited contextualized evidence in Liberia or similar contexts. Additionally, the study participants noted that critical topics were not fully incorporated into the policy, including strong gender equality and human rights. Gender affirmative actions that could have ensured the equitable representation of men and women among CHAs were not well articulated in the policy. Also, the policy design prioritized formalizing CHAs in the workforce, but did not sufficiently integrate the existing community health volunteers, including TTMs. Instead, this complementarity emerged organically in the community, when the community members sought TTMs support for sexual and reproductive health, pregnancy and delivery. According to the interviews, TTMs, whom are all women, had strong relationships with the communities they served, particularly women, and making health service provision more gender responsive.

### Community dynamics and root causes of the gender inequitable CHA selection

Despite the written guidance of the RNCHSP 2016–2021 to prioritize the selection of women as CHAs, the study confirmed that the community-led nomination and selection process did not result in this outcome. Instead, men are overrepresented in the nomination of CHAs as neither the RNCHSP nor the NCHAP provided the communities with sufficient guidance in how to prioritize women in the nomination and selection process.

At the community level, CHCs, which are made up of key community members, are responsible for the nomination and selection process of CHAs. Despite the strong presence of women in the CHCs, most CHCs did not prioritize the nomination of women as CHAs due to community gender norms and practices. For example, the interviews revealed that CHCs prioritized the nomination of men because of their perception that men are more entitled to remunerated jobs. More than half of the study participants stated that harmful traditional, religious and cultural norms restricting women to household responsibilities were barriers. These household duties included childcare and cooking for the entire family, while men focused on income generating work. Similarly, women’s lack of autonomy in decision making emerged as a critical barrier to being nominated and selected as a CHA.

One study participant noted:

At the initial stage of the selection of CHAs, all these information was given to the community, but during the selection or election process, the town or the community chose to use men rather than women, so in most of the places you will find out that women were denied, or women were not selected… CHFP (Man)

Another key barrier women face in becoming CHAs is the requirement in the RNCHSP for CHAs to have a minimum equivalent of a 6th grade education. The findings highlight that women and girls in Liberia have limited access to education and low educational attainment due in part to family preference in selecting a male child to attend school and other harmful traditional practices such as child marriages. Further, women were not nominated or selected into the NCHAP on the grounds of being perceived by others and self-described as shy and not confident. The community perceptions of women’s competency, and consequently women’s nomination, do not consider the limited opportunities and larger barriers women face compared with men. The community selection process envisaged by the policy did not consider women’s barriers to educational attainment or literacy, given educational attainment was a core CHA nomination and selection criteria. One participant noted the impact of traditional and cultural norms in influencing women and girls’ education:

Yes, in our culture especially where the programme has been implemented in rural parts of Liberia, everybody in their own household have their own rules and responsibilities… They prefer their wives to stay home, wash clothes and look after the children… So, each man or woman has their role to play for every household. Also, there are roles for the children where in the rural setting, they will prefer the boy child leaving from their community to go out to learn while the girl child stays there and learn from their mother how to take care of their home. CHFP (Man)

This study also highlighted the structural challenges of implementing RNCHSP that affect both men and women CHAs. For example, timely remunerations, sustainable supply chain of commodities, long distances to the communities and motivation. However, the study participants suggest that the impact is likely to affect women more, as explained in the next section.

### Roles and responsibilities of CHAs and gender preference of CHAs by community

The primary beneficiaries of the CHAs services are women and children receiving reproductive, maternal, neonatal and child health care services. Women stated they preferred to receive services from women CHAs because they exhibit more patience and empathy compared with men. Other interviewees noted that women in the community are uncomfortable confiding in and do not trust CHAs who are men about reproductive health matters, such as pregnancy and menstruation. This preference by women in the community set the foundation for a generalized lack of acceptability and accessibility of services provided by CHAs who are men. In addition, the study showed that when women receive services from CHAs who are men, their husbands were afraid of infidelity. The limited domestic trust is suggested to impact women’s access to health services.

The study also highlighted that the RNCHSP envisaged the same job responsibilities for CHAs regardless of their gender. However, when implemented, roles and responsibilities were differentiated by a gendered division of tasks and social constructs of gender. For example, CHAs that are women attended to reproductive and maternal health needs, including pregnancy-related health outcomes for above-mentioned reasons, whereas men attended to other health needs.

Further, neither the policy nor programme implementation includes gender-responsive measures for transportation, safety and logistics leaving women to experience more hardships. This challenge emerged clearly among CHSSs who supervise CHAs over large geographic areas. While few CHSSs may utilize motorbikes, CHSSs that are women were less familiar with maintenance and repairs.

The lack of security for CHAs limits their ability to provide services when called at night or into more remote areas. Risk of gender based and ritual violence also disproportionally affect women. One CHSS felt that these safety issues affect women CHA’s ability to execute their tasks effectively.

if it is a female you alone you will not be able to walk that distance to go to that places because the ritualistic killing is too much on this side so people don’t just move alone so when it is a female, it will be difficult for you. In that case now to do the work it will be another setback to you for your job. CHSS (Woman)

### Consequences of inequitable gender ratio among CHAs

The study identified the consequences of gender inequity in the NCHAP across two major dimensions: the health workforce and service delivery. The study observed an increasing reliance on TTMs, who are all women, to accommodate the cultural sensitivity and community preference for women service providers, particularly in providing reproductive and maternal health services. The gender imbalance in the RNCHSP and its programme implementation also affected the affordability of healthcare services, with some communities spending additional out of pocket expenditures to access the gender-sensitive services offered by TTMs.

### Promising gender responsive solutions

The key informants identified a few interventions to overcome the gender imbalance among CHAs in Liberia. Some organizations in Liberia have piloted a pairing approach to combine CHAs of both genders to enable better access and acceptance to the community. Some interviewees suggested pairing TTMs with CHAs and others suggested pairing couples (husband and wife) to overcome the cultural and gender barriers. Most study participants identified affirmative actions, such as a gender quota among CHAs, as possible effective interventions, particularly if clearly articulated and integrated into the policy. Some interviewees highlighted the promising results of working with the communities to enforce positive gender norms and address negative gender norms and harmful practices. This community approach is perceived to address the root causes of gender imbalance among CHAs. Interviewees recommended improving educational opportunities for women since many women do not meet the education eligibility criterion. Some NCHAP implementing partners are exploring partnerships to create a career ladder for graduated young women to pursue opportunities as CHAs. Also, a few implementing partners highlighted how the digitization of tools for monitoring and evaluation (M&E) has the potential to strengthen the gender responsiveness of community health activities, given that visual aids on tablets or mobile phones could enable less literate users to perform the same CHA duties. However, these interventions were not formally evaluated or appraised.

## Discussion

**Figure 2.**
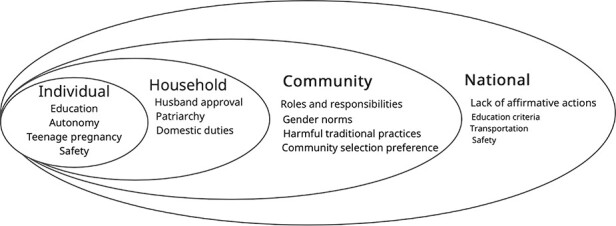
Socioecological model

This study represents the first systematic examination of gender responsiveness among the community health workforce in Liberia. The study offers in-depth analysis of the contextual drivers, challenges and opportunities in developing both policies and programme in Liberia. Despite being conducted in only five counties, the findings on policy and structural barriers are largely generalizable nationwide, given the representative sample of counties chosen. Also, it contributes to the global evidence that gender dynamics influence CHW selection and their roles and responsibilities (Raven *et al.*, 2020). Further, the study shows how the gender dynamics in a CHW programme impact women’s access to primary healthcare, including maternal and reproductive health (Feldhaus *et al.*, 2015). The study found that the RNCHSP 2016–2021 was not gender responsive, resulting in limited gender responsiveness in programme implementation. Specifically, the study found that the CHA selection process at the community level did not result in the intended gender ratio balance of CHAs. Consequently, communities experienced limited access to CHA services—especially women in the community who preferred for their CHA to be a woman but were served by a man. Following the socioecological model as illustrated in Figure 2, the study appraises the impact of gender dynamics on CHAs at the individual, household (interpersonal), community and national levels (Steege *et al.*, 2020; Dahlberg and Krug, 2006).

### National level

Findings from this study align with the emerging global evidence that women’s participation in the community health workforce depends not only on the gender responsiveness of the health system but also on the gender norms within the community (Steege *et al.*, 2018). Therefore, a paradigm shift in community health policies is needed to move away from implicitly cultural norms that favour men, particularly in low and middle income countries (Steege *et al.*, 2018).

The study participants recommended developing proactive policies that incorporate gender affirmative actions in selecting and recruiting CHWs, particularly in contexts where gender norms, traditions and inequalities are substantial barriers, in line with the World Health Organization guidelines (WHO, 2018). Recent evidence suggests moving away from a non-gender-responsive approach where community health policy interventions and activities are designed without considering the different social constructs of genders and their different needs (Mckague and Harrison, 2019). Instead, community health policies should be designed with a gender-responsive approach that proactively identifies and examines gender inequalities, gender-based constraints and inequitable norms and dynamics (Mckague *et al.*, 2021).

This study shows the impact of the policy and programme that were not designed with a gender-responsive approach. The study identified several gender-related challenges affecting women CHAs not considered in the policy design, including safety concerns related to travelling remote distances, lack of transportation, remuneration and other related logistics. Enabling better working conditions is believed to improve the performance and retention of women CHAs/CHSSs. These working conditions take gender-responsive actions in facilitating women’s safety, transportation and duties. In addition, measures like maternity leave and sick pay can assist female CHWs in remaining in employment (Steege *et al.*, 2018).

At the national level, women and girls are excluded from the RNCHSP and NCHAP by educational eligibility requirements. These findings are consistent with findings from similar contexts (Raven *et al.*, 2020). Participants from this study suggested developing a partnership with the Adult Education Programme and the Ministry of Education in Liberia to facilitate progressive learning for out of schoolgirls and women. However, there is limited empirical evidence on the effectiveness of this approach.

While the policy in Liberia recommended the remuneration of all CHWs, significant challenges exist in sustaining remuneration for both male and female CHWs in sub Saharan Africa. In Rwanda and Kenya, the lack of compensation for CHWs perpetuates the continued harmful gender norms that consider women’s labour as free of charge (Newman *et al.*, 2011; Kok *et al.*, 2015). For example, the lack of remuneration in a programme that promotes women’s involvement only contributes to women’s work being undervalued by society (Newman *et al.*, 2011). Further, despite payment not being the only motivation for CHWs, the lack of pay may also contribute to poor retention and motivation (Raven *et al.*, 2015). Severe community health funding bottlenecks exacerbate these challenges (Greenspan *et al.*, 2013; Simen-Kapeu *et al.*, 2021).

The study participants showed interest in exploring pairing techniques, with CHAs that are men being accompanied by TTMs, as a cultural- and gender-responsive approach to reach communities. However, this approach should only be considered if TTMs are formally included and remunerated in the policy, otherwise it will exacerbate the accessibility of services if communities continue to pay TTMs out of pocket and further devalue the work done by TTMs The regional evidence suggests that CHW pairing techniques of deliberately pairing a man and woman has improved communication and contributed to the equitable access of community health services for men and women. Pairing allows the teams to provide care based on the acceptance and preference of the household, to mitigate the community’s limited acceptance of CHWs who are men, especially in sexual and reproductive health and ensure that men can access community health services, as piloted in Kenya (Mckague and Harrison, 2019).

A dual cadre model was implemented in Ethiopia whereby village volunteers complement the highly trained CHWs (Schleiff *et al.*, 2021). This model may compensate for the shortage of healthcare workers and offer gender balance, particularly in contexts with high literacy rates among women and girls. However, the dual cadre approach may lead to unintended consequences by framing women and girls as volunteers, likely to receive less financial remuneration and a professional career ladder (Steege *et al.*, 2018).

Moving towards gender-responsive policies requires systematic gender analysis of the health workforce and addressing gender-based constraints for women and men by employing innovative strategies for improved gender equity (Mckague and Harrison, 2019). For example, recent research across Africa reiterated the importance of career pathways for CHWs that are women, impacting their self-confidence and self-perception as legitimate work peers to their male colleagues and communities. Therefore, community health policies should establish clear rewards and pathways for promotion (Mckague and Harrison, 2019). This study found that RNCHSP and NCHAP did not powerfully articulate a career ladder or leadership prospects. Additionally, the study highlighted the importance of dedicating a gender quota to the middle and senior management positions and the positive impact it could have on gender equity among women at all levels of decision making (Mckague and Harrison, 2019).

The study showed that the utilization of a multisectoral approach in policy design can improve health outcomes, optimize financial and human resources of the different sectors and address the social determinants of health that are rarely addressed in health only policies. The use of multisectoral programming in health across similar contexts is believed to contribute to better health outcomes (Bennett *et al.*,2018).

### Community level

At the community level, reduced access to education and low educational attainment for girls and women prevented their selection into the RNCHSP and NCHAP. Most women who did not demonstrate the ability to read and write, add, subtract and multiply in English and complete a literacy test did not make it in the CHA recruitment process. In Liberia, a significant contributor to girls’ and women’s lack of education is the families and communities’ preference to send boys to school and have girls stay home, an argument opposed by Longfield and Tooley (2017). Addressing barriers to girls’ education requires implementing successful gender transformative approaches at the community level that include challenging patriarchal hierarchies (UNFPA/UNICEF, 2020).

Further, the community nomination and selection process favoured men over women despite the RNCHSP and NCHAP stating that preference should be given to women in the CHA nomination process (Steege *et al.*, 2020). The monetization of the CHA role may have contributed to this male preference, thus deeming it as being meant for men by community members. Other reasons may be attributed to patriarchal norms that inhibit women’s ability to take up significant roles in the community and restrict them to household duties. However, evidence shows that the payment of female CHWs, such as Health Extension Workers in Ethiopia, provides economic independence and a source for women’s empowerment (Closser *et al.*, 2019; Steege *et al.*, 2020). The need for the communities to challenge patriarchal norms of men deciding whether female household members can work outside the home (USAID, 2018; Steege *et al.*, 2020).

## Household level

The findings demonstrate how men often used traditional, cultural and religious norms to reduce women’s autonomy in seeking health care or working as CHAs. Research from Namibia, Nepal, Kenya, Pakistan and India also found that men play a role in barring their wives from working in CHW programme (Mumtaz *et al.*, 2003; Olang’o *et al.*, 2010; Alam and Oliveras, 2014). Further, our study corroborates other literature that shows that a woman’s access to CHW services was affected by men in the household. In Liberia, husbands prevented their wives from accessing services from CHAs who men are out of fear of infidelity, creating a barrier to healthcare services, including maternal and reproductive health services. This contributes to preventing women from fully realizing their health and wellbeing, as evidenced by the high rates of poor reproductive and maternal health outcomes in Liberia (Namasivayam *et al.*, 2012; Lisgis, 2021).

### Individual level

In the case of education, which is a critical selection criterion for the CHA programme, girls in Liberia remain disadvantaged compared with their male counterparts on all education indicators, including literacy rates, gross enrolment and school completion rates at both primary and secondary levels (UNESCO, 2022). Therefore, educational attainment and illiteracy were major barriers to recruiting women CHAs. These findings align with regional findings from a study in DRC, Sierra Leone and Liberia that recommended providing basic literacy and numeracy training for women and girls to qualify them for the selection criteria of CHAs (Raven *et al.*, 2020).

This study shows that early adolescent girls’ sexual and reproductive health outcomes can negatively impact their future education and economic prospects. The high rate of adolescent pregnancies in Liberia affects school completion, contributing to girls not living to their fullest potential. In the case of CHAs in Liberia, girls and women have not been selected for the RNCHSP and NCHAP due to limited educational attainment that could have resulted from school dropping out due to early pregnancy (Lisgis, 2021, Nichols, 1987). Besides education, women’s limited autonomy in decision making and confidence to pursue a career as CHAs emerged as another barrier.

Safety was another challenge for women executing their duties as a CHA, with the heightened risk of being raped or sexually harassed (Uzondu *et al.*, 2015). This study aligned with the global literature on how safety is a challenge for women CHWs and protecting women from these risks needs to be built into programme design and implementation.

The study found that the male dominated CHA workforce affected how women accessed health services in Liberia. Liberian women, particularly pregnant women, showed a strong preference to be served by female CHAs. Consequentially, the male dominated NCHAP reduced access to reproductive and maternal health services, consistent with past research (Feldhaus *et al.*, 2015). The data did not indicate the impact of a male dominated NCHAP on men’s access to care.

### Implications and what’s next?

In order to implement a gender-responsive NCHAP, interventions are required to address challenges at the individual, household, community and national levels, including close collaboration among the Ministries of Health, Gender and Internal Affairs. The findings from this study provide an opportunity to strengthen the community engagement, local ownership, formal and informal coordination mechanisms at all levels. A summary of the policy and programme implementation recommendations are provided in Table 2. Rigorous design, piloting and evaluation of the viability of these recommendations within the Liberian context is needed to build stronger evidence the effectiveness of these interventions.

**Table 2.** Summary of actionable recommendations to strengthen the gender responsiveness of the RNCHSP and NCHAP

Thematic areas	Policy and programme recommendations
Affirmative actions	Include a gender quota among CHAs and similar positions in the policy. At the implementation level, review the country-level gender ratio and prioritize gender affirmative actions in counties with the least women CHA gender ratio.
Multisectoral approach in policy design	Engage line ministries (such as the Ministry of Gender and Protection), key gender experts, implementing partners and bilateral donors, throughout policy development rather than only at the review stage.
Community engagement and sensitization	Promote multisectoral CHA programming that integrates gender to address social, cultural and gender norms and harmful traditions. Joint programme with the Ministry of Gender, Ministry of Youth and Sports and Ministry of Internal Affairs can effectively mobilize resources and efforts and deliver strong messages on gender equity within the communities and among CHAs.
Education and adult education	Given that education was a significant barrier to the gender-equitable ratio among CHAs, adult education programme should be offered as a pathway for female graduates to join the CHA programme. Further, MOH should collaborate with the Ministry of Education to offer a career ladder for school graduates to work as CHAs within their communities.
Pairing techniques	To address the gender imbalance among CHAs, the RNCHSP and NCHAP should consider pairing techniques. Men CHAs are accompanied by women TTMs to improve access to service delivery to key communities. Fair financial compensation and formalization of TTMs roles in community health should be clearly articulated in the policy.
Digitalization	The digitalization of community health tools and documents can enable women with limited literacy to log data, report and manage their tasks better than the paper-based work modalities. Thus, contributing to relieving the educational requirements for CHAs. Digital health technologies can also provide healthcare services where KII CHAs cannot provide in-person services.
Gender-sensitive data	Assign gender-sensitive targets and indicators to monitor the gender dimension in the regular policy and programming review processes.
Evidence generation	Identify evidence gaps and how they can be addressed by the collective efforts of the line ministries, implementing partners and donors. Key evidence gaps include how the gender of the community health worker impacts health outcomes, across different counties. Also, future studies on the gender dynamics and decision making of the CHCs to develop gender-responsive selection should be conducted.

## Limitations

Despite the many strengths, the study exhibits several limitations. The limited literature compromises the robustness of the literature and desk review design on the Liberian context. In addition, limited counterfactuals, constraints in identifying the sound basis for comparison, limited quantitative data and possible biases of key stakeholders represented key challenges in the analysis. Despite conceptualizing gender in a nonbinary definition, the analysis drew on the binary categorization of gender as shared by most key informants. The findings of the gender assessment are limited by the inability to hold additional interviews from different line ministries and in the districts.

### Recommended research

Future research can investigate the gender dynamics during the community selection process to establish who was present at these meetings and what role different genders played during these processes. Researchers should be encouraged to use innovative methods, including mixed methods, affordability and economic costing. It can also investigate and determine the risk of gender-based violence and general safety experienced by women CHAs when conducting their duties.

## Conclusion

Gender inequity in Liberia is rooted in various structural and social cultural norms and perceptions. This systemic inequity permeates through societal structures, including the community health workforce, applying a gender transformational approach that prioritizes the needs of women CHAs and community members in policy design and implementation. This approach challenges inequitable gender norms and creates a more gender-responsive RNCHSP and NCHAP. Employing a human rights based and community centred approach that addresses the health needs of the communities is crucial for developing equitable health policies and contributing to advancing UHC and the sustainable development goals.

## Abbreviations

CHAs = Community Health Assistants

CHCs = Community Health Committees

CHFP = Community Health Focal Person

CHSSs = Community Health Services Supervisors

CHVs = Community Health Volunteers

CHWs = Community Health Workers

gCHVs = general Community Health Volunteers

HFDCs = Health Facility Development Committees

INGOs = International Non-Governmental Organizations

KIIs = Key Informant Interviews

MOH = Ministry of Health

M&E = Monitoring and evaluation

NCHAP = National Community Health Assistant Programme

NGOs = Non-Governmental Organizations

RNCHSP = Revised National Community Health Services Policy (2016–2021)

TTMs = Trained Traditional Midwives

UN = United Nations

UHC = Universal Health Coverage.

## Reflexivity statement

The majority of study co-authors are from the global south (9 out of 11). Of them, five are Liberians, including the principal investigator. We, all co-authors, had in-depth knowledge and prior work experience in Liberia or West Africa.

During the research process, the data collection team (SH, BM, MJ and MK), paid attention to our own beliefs, judgements and practices and reflected upon how these may influence our understanding of the data and management of the study. We relied heavily on the experience of the Liberian co-authors and the Liberian Advisory Gender Expert Panel to align our potential differences in perspectives and gain a deeper understanding of the data. All the data collected with the communities and community health workers were collected by a Liberian researcher to ensure that nothing was lost in interpretation or translation when the researchers delivered analysis data from Liberian pidgin English to UK English. All the contributing co-authors who identify as men, recognise their gender privileges and remained reflective of all potential biases when conducting the Gender Assessment of the NCHAP.

## Appendix 1. The study team

The majority of study co-authors are researchers and public health specialists from the global south (9 out of 11). Of them, five are Liberians, including the principal investigator (MS). We, all co-authors, had in-depth knowledge and prior work experience in Liberia or West Africa.

During the research process, the data collection team (SH, BM, MJ and MK), paid attention to our own beliefs, judgements and practices and reflected upon how these may influence our understanding of the data and management of the study. We relied heavily on the experience of the Liberian co-authors and the Liberian Advisory Gender Expert Panel to align our potential differences in perspectives and gain a deeper understanding of the data. All the data collected with the communities and community health workers were collected by a Liberian researcher to ensure that nothing was lost in interpretation or translation when the researchers delivered analysis data from Liberian pidgin English to UK English. The study early findings were reviewed and validated by representatives from all the counties in Liberia. All the contributing co-authors who identify as men, recognize their gender privileges and remained reflective of all potential biases when conducting the gender assessment of the NCHAP.
